# Characterization of sebaceous and non-sebaceous cutaneous manifestations in patients with lynch syndrome: a systematic review

**DOI:** 10.1007/s10689-022-00319-8

**Published:** 2022-11-23

**Authors:** Shahram Aziz, Hazel O’Sullivan, Kara Heelan, Afrina Alam, Terri P. McVeigh

**Affiliations:** 1 Department of Physiology, University of Sulaymaniyah, Sulaymaniyah, Iraq; 2 The Royal Marsden NHS Foundation Trust, London, UK; 3 Department of Dermatology, Imperial College London & Chelsea and Westminster Hospital, London, UK; 4 Komar University of Science and Technology, Sulaymaniyah, Iraq

**Keywords:** Lynch syndrome, Muir-Torre syndrome, Mismatch repair, Sebaceous tumour; squamous cell cancer; basal cell cancer

## Abstract

A subset of patients with Lynch Syndrome demonstrates cutaneous manifestations of the disorder. Characterization of these Lynch-related skin lesions could help in early recognition of patients with Lynch Syndrome. A broad search of the literature on OVID Medline and Embase was carried out to capture papers reporting cutaneous manifestations in Lynch Syndrome patients. The results were uploaded into Mendeley reference management software. The PRISMA workflow was used in the literature selection process. In this systematic review, data were collected from 961 cases from 413 studies, including 380 molecularly confirmed Lynch Syndrome cases. The main skin lesions were: Sebaceous adenomas (43%), sebaceous carcinomas (27%), keratoacanthomas (16%), sebaceomas (13%), squamous cell carcinomas (23%), and basal cell carcinomas (10%). *MSH2* variants were the most common underlying genotype (72%). Assessment of mismatch repair by immunohistochemistry, microsatellite instability analysis, or both were performed on 328 skin lesions from 220 (58%) molecularly confirmed cases. In those skin lesions, 95% of Immunohistochemistry and 90% of the microsatellite instability test results were concordant with the underlying genotype. Sebaceous skin lesions are well-recognised phenotypic features of Lynch Syndrome. Our results show that squamous and basal cell carcinomas are relatively common in patients with Lynch syndrome; however, available evidence cannot confirm that Lynch syndrome is causal. Immunohistochemistry and/or microsatellite instability testing of skin tumours in patients with a family history of Lynch Syndrome-associated cancers may be a useful approach in identifying patients requiring referral to Clinical Genetics and/or consideration of germline genetic testing for Lynch Syndrome.

**Supplementary Information:**

The online version contains supplementary material available at 10.1007/s10689-022-00319-8.

## Introduction

Lynch Syndrome (LS), formerly known as hereditary non-polyposis colorectal cancer syndrome, is a common hereditary cancer predisposition syndrome with an estimated prevalence of 1 in 300 [1]. It is an autosomal dominant disorder most commonly caused by constitutional pathogenic variants in one of four mismatch repair (MMR) genes: *MLH1*, *MSH2*, *MSH6* and *PMS2*. In approximately 1–3% of families, LS can be caused by constitutional deletions in the 3′ end of *EPCAM*- leading to hypermethylation and transcriptional silencing of *MSH2*. A small proportion of LS is caused by a de novo or inherited constitutional epimutation of *MLH1* [1, 2]. LS is associated with an increased lifetime risk of cancers of the colorectum, endometrium, ovaries, stomach, small bowel, bile duct, pancreas, and upper urinary tract [1, 2]. Cutaneous tumours represent 3–5% of extra-colonic malignancies of LS [3–5].

Based on reported population frequencies of constitutional pathogenic MMR gene variants, it is estimated that approximately 175,000 people in the UK have LS, but diagnosis has only been confirmed in approximately 5% [1, 6]. Confirming a diagnosis of LS in patients with cancer is important to guide therapeutic decision-making, inform the risks of subsequent cancers, facilitate early detection and/or cancer prevention, and allow for predictive genetic testing of at-risk relatives [1, 6]. Cancers occurring within the context of LS typically demonstrate Mismatch repair deficiency (MMRd), with loss of one or more MMR proteins detectable by immunohistochemistry (IHC), although MMRd is more commonly caused by sporadic somatic events [1, 7]. Mismatch repair deficient tumours, whether due to germline or somatic events, also typically demonstrate microsatellite instability (MSI) [1, 7]. To help improve identification of patients with LS, guidelines from the National Institute for Health and Care Excellence (NICE) have been issued recommending assessment of colorectal and endometrial tumours by IHC or MSI testing, with subsequent germline MMR gene testing as required [1, 7]. Furthermore, a National LS Project has been established through the NHS Genomic Medicine Centres Alliance to help implementation of these guidelines, with plans to expand testing to non-colorectal/non-endometrial LS-associated cancers.

Sebaceous neoplasms and keratoacanthomas occur as part of a well-recognised phenotypic variant of LS, known as Muir-Torre syndrome (MTS) [8, 9]. Other types of reported cutaneous neoplasia occurring within the context of LS include basal cell carcinomas (BCC) and squamous cell carcinomas (SCC) [10–13]. It has not yet been determined if the risk of non-sebaceous cutaneous malignancies is attributable to patients’ underlying genotype. In this systematic review, we sought to critically evaluate the published literature regarding the frequency and characteristics of sebaceous and non-sebaceous cutaneous manifestations in patients with LS. We also aimed to provide data describing the outcomes of MMR protein immunohistochemistry, MSI testing, and germline genetic testing in such cases.

## Patients and methods

### Search strategy

A systematic review was undertaken following the Preferred Reporting Items for Systematic Reviews and Meta-Analyses (PRISMA) workflow. To identify articles relevant to the study topic, a broad search of the literature was first conducted, after which, identified papers were manually screened using various criteria. The key terms used for the literature search were selected after a preliminary search of both medical subject headings and free-text terms performed on Google and Ovid. This preliminary search produced fifteen key terms that were deemed relevant to the topic, namely “Lynch syndrome”, “skin”, “cutaneous”, “derm*”, “sebaceous”, “sebaceoma”, “keratoacanthoma”, “Muir Torre Syndrome”, “hereditary nonpolyposis colorectal cancer”, “HNPCC”, “squamous cell”, “SCC”, “basal cell”, “BCC”, and “melanoma”.

The literature search was performed on January 15th, 2022, using Ovid to extract articles from the Medline and Embase databases. To fully capture all relevant articles, an advanced search was conducted using the aforementioned key terms individually (Muir Torre syndrome) or in the following combinations: (Lynch syndrome and skin), (Lynch syndrome and cutaneous), (Lynch syndrome and derm*), (Lynch syndrome and sebaceous), (Lynch Syndrome and sebaceoma), (Lynch Syndrome and keratoacanthoma), (Lynch Syndrome and squamous cell), (Lynch Syndrome and SCC), (Lynch Syndrome and basal cell), (Lynch Syndrome and BCC), (Lynch Syndrome and melanoma), (hereditary nonpolyposis colorectal cancer and skin), (hereditary nonpolyposis colorectal cancer and cutaneous), (hereditary nonpolyposis colorectal cancer and derm*), (hereditary nonpolyposis colorectal cancer and sebaceous), (hereditary nonpolyposis colorectal cancer and sebaceoma), (hereditary nonpolyposis colorectal cancer and keratoacanthoma), (hereditary nonpolyposis colorectal cancer and squamous cell), (hereditary nonpolyposis colorectal cancer and SCC), (hereditary nonpolyposis colorectal cancer and basal cell), (hereditary nonpolyposis colorectal cancer and BCC), (hereditary nonpolyposis colorectal cancer and melanoma), (HNPCC and skin), (HNPCC and cutaneous), (HNPCC and derm*), (HNPCC and sebaceous), (HNPCC and sebaceoma), (HNPCC and keratoacanthoma), (HNPCC and squamous cell), (HNPCC and SCC), (HNPCC and basal cell), (HNPCC and BCC), and (HNPCC and melanoma). At a later stage, the screened papers’ reference lists were manually reviewed to identify further relevant articles.

### Inclusion criteria

All papers reporting studies that provided relevant primary or secondary data were considered for inclusion in this review regardless of date and language of publication. All patients with clinically (Amsterdam and/or Bethesda criteria) or molecularly (following germline testing) confirmed LS and skin lesions, and all patients meeting the clinical criteria for MTS (sebaceous neoplasms or multiple keratoacanthoma, and visceral malignancies) were included.

### Data extraction

To ensure consistency and structure in the extraction of the data from the studies included in this review, a data extraction form was used. This form was adapted from the Data Extraction and Assessment Form provided by the Cochrane good practice data extraction guidelines and was formatted for fast and effective extraction of the type of data needed. The form was first piloted on 20 randomly selected papers, further edited, and then standardized. Cases were utilized as the unit of measurement to determine the frequency of skin lesions. The pathogenicity of reported variants was assessed in brief by reviewing reported classifications in ClinVar and InSIGHT databases. Studies published in non-English languages were translated using Google translate, and through native speakers when necessary.

### Data analysis

The quantitative results are presented using descriptive statistics. The Statistical Package for the Social Sciences (SPSS) version 25 and Microsoft Excel were used to produce all the statistics. The distribution of quantitative data was assessed using the Kolmogorov–Smirnov test, and parametric or non-parametric tests applied as appropriate. P value of ≤ 0.05 was considered statistically significant.

## Results

### Study selection

**Fig. 1:**
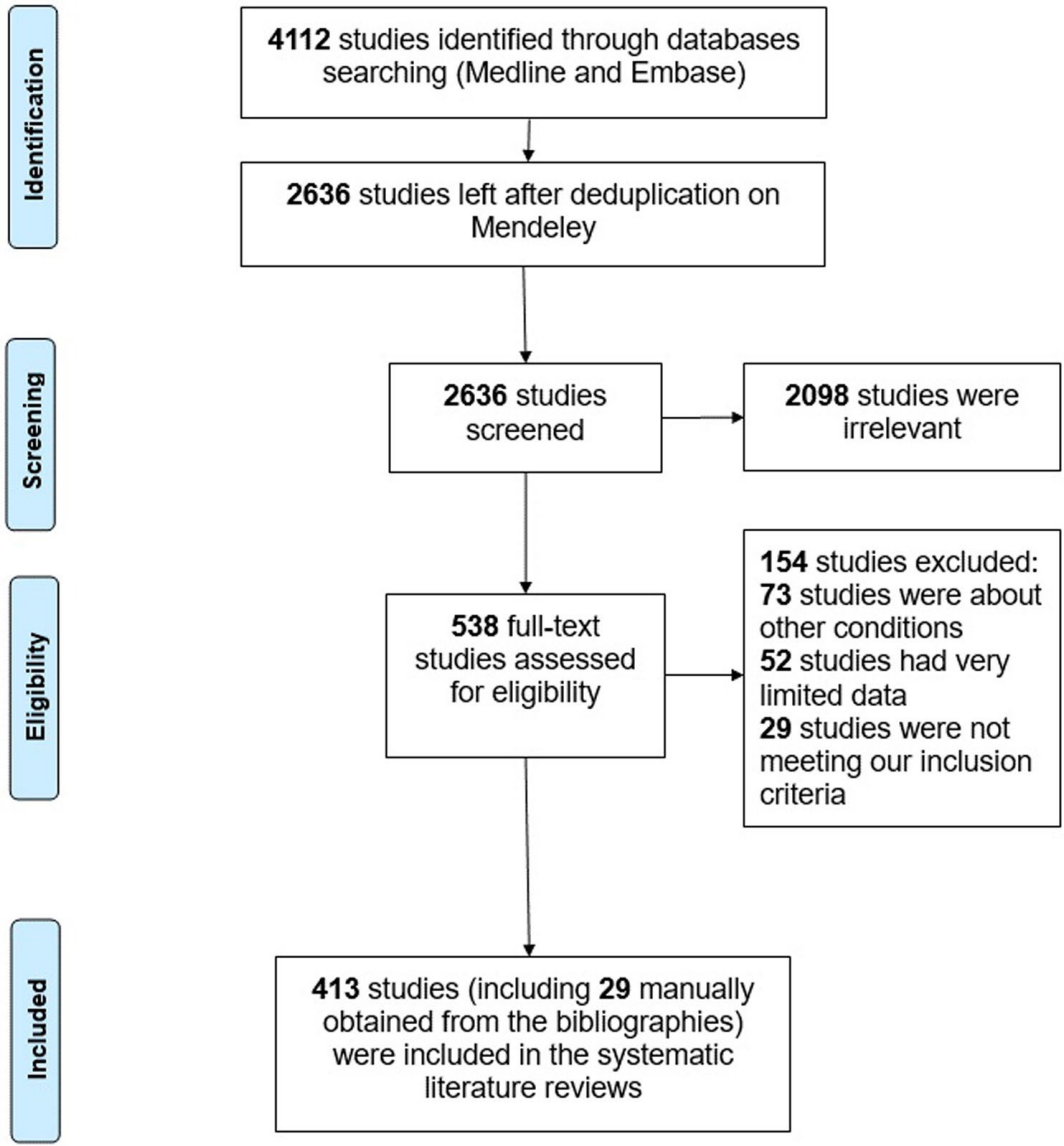
Flowchart showing the literature selection process following PRISMA guidelines

Our literature search produced 4112 papers in the initial screening phase. After duplicate papers were removed, 2636 papers remained. We performed title and abstract screening which led to a further 2098 papers being eliminated, leaving 538 papers for the full-text screening. These papers were further reviewed, and data was extracted from 413 papers in this literature review. Figure 1 shows the study selection process and the results of the literature search.

### Summary of included studies

Publications with relevant information were included regardless of the study type, date, location, and language of publication. 279 (68%) of the papers were case reports and 134 (32%) case series (Online Resource 1).

### Patient characteristics

Four hundred and thirteen papers were included, from which data was described regarding 961 patients. Among them, 776 patients (81%) fulfilled clinical diagnostic criteria for Muir-Torre Syndrome. Five hundred and eighty-one patients (60%) were included based on: history alone (n = 328) or history and tumour IHC and/or MSI results (n = 253). Germline genetic testing was undertaken in 432 patients (44%), of whom 380 (90%) had molecular confirmation of a diagnosis of LS.

Most (202, 65%) of the patients had multiple (≥ three) skin lesions, and the majority of patients (321, 85%) had sebaceous lesions. Non-sebaceous skin lesions were more frequently located in sun-exposed areas compared to sebaceous lesions (71%-v- 57%, p = 0.01, *X*^2^) (See Table 1).

**Table 1:** Demographics and characteristics of molecularly confirmed cases:

Variable	N (%)
Gender	
Male	140 (37%)
Female	89 (23%)
Unreported	151 (40%)
Ethnicity	
Caucasian	27 (7%)
Asian	7 (2%)
Ashkenazi Jew	4 (1%)
African	4 (1%)
Arab	1 (1%)
Unreported	337 (88%)
Type of skin lesion	
Sebaceous	218 (58%)
Non-sebaceous	53 (14%)
Both	100 (26%)
Unreported	9 (2%)
Number of skin lesions per patient	
One	84 (22%)
Two	23 (6%)
> Three	202 (53%)
Undetermined	71 (19%)
First type of cancer in patient	
Cutaneous	76 (20%)
Non-cutaneous	143 (38%)
Unreported	161 (42%)
Family history of cutaneous malignancy	
Sebaceous	19 (5%)
Non-sebaceous	41 (11%)
No reported family history of cutaneous disease	173 (45%)
Unreported	147 (39%)
Median age at diagnosis of cutaneous lesion	
Sebaceous adenoma	55 (28–77)
Sebaceous carcinoma	56 (31–84)
Sebaceoma	56 (41–73)
Keratoacanthoma	52 (34–70)
Squamous Cell Cancer	55 (33–75)
Basal Cell cancer	53 (29–68)

### Germline genetic testing

**Fig. 2:**
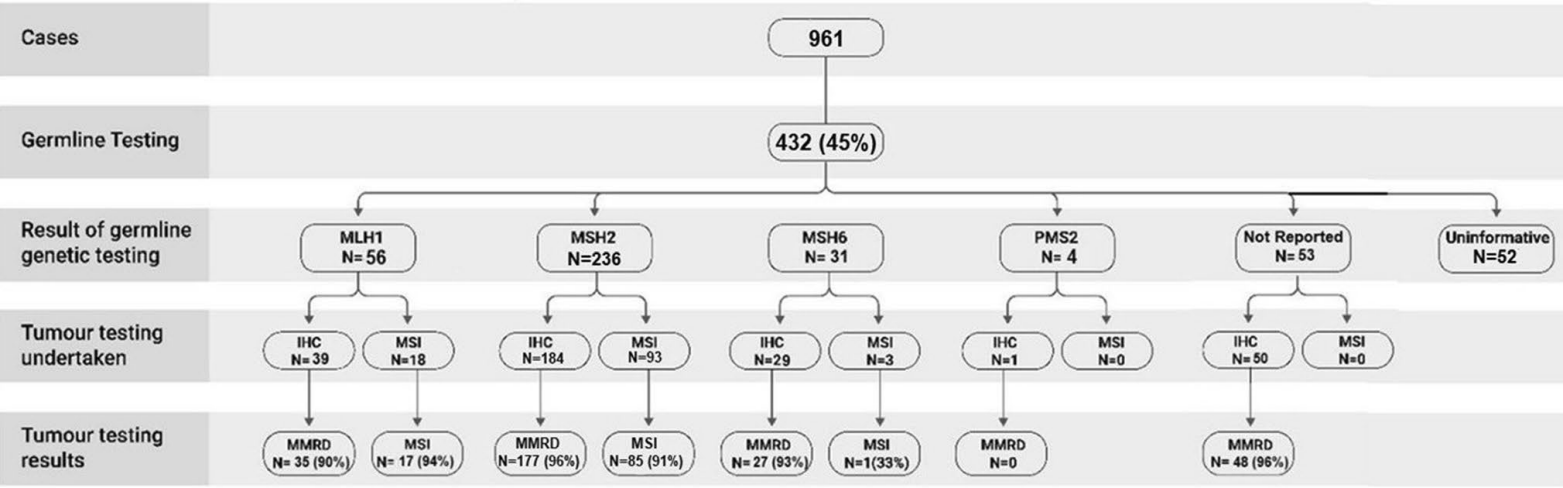
Flowchart showing the number of cases included in this review, their genotypes, and results of immunohistochemistry and microsatellite instability testing of skin lesions

Germline testing was performed in 432 patients (Fig. 2). In these cases, the tests were prompted by patients’ histories alone (n = 144, 33%), history and IHC (93, 22%), or MSI (33, 7%) or both (111, 26%), and undetermined in 51 (12%). Germline testing provided molecular confirmation of LS in 380 (88%) cases, while 41 (12%) cases had uninformative tests. Pathogenic variants were most commonly identified in *MSH2* (n = 236, 62%), followed by *MLH1* (n = 56, 15%), *MSH6* (n = 31, 8%), and *PMS2* (n = 4, 1%) genes. The name of the gene was not reported in 53 (14%) patients. The specific genetic defects or variants were reported in 235 cases (62%). The pathogenic variant (NM_000251.3(*MSH2*):c.942 + 3A > T) was the most commonly reported single variant, reported in 18 cases (8%) from 17 families in 9 studies. The complete list of reported variants is shown in (Online Resource 2). Among those cases with uninformative test results, the extent of germline genetic testing was variable, with a one-third having testing of four MMR genes (n = 18, 35%). Testing of *MSH2* only was performed in 1 case (2%), of *MLH1* and *MSH2* in 17 cases (33%), and of *MLH1*, *MSH2*, and *MSH6* in 10 cases (19%). Extent of testing was not specified in 6 cases (11%). The details of the cases with uninformative germline testing are shown in (Online Resource 3). The frequencies of skin lesions according to their genotypes are shown in Table 2.

**Table 2:** Skin lesions according to the underlying genotype of affected patients:

Genotype of affected cases	Total no. of cases	Cases with SA	Cases with SC	Cases with sebaceoma	Cases with KA	Cases with SCC	Cases with BCC
*MSH2*	236	106 (45%)	62 (26%)	34 (14%)	47 (20%)	55 (23%)	24 (10%)
*MLH1*	56	21 (38%)	18 (32%)	7 (13%)	4 (7%)	13 (23%)	5 (9%)
*MSH6*	31	11 (35%)	8 (26%)	3 (10%)	1 (3%)	6 (19%)	3 (10%)
*PMS2*	4	3 (75%)	–	–	–	–	–
Total	327	141 (43%)	88 (27%)	44 (13%)	52 (16%)	74 (23%)	32 (10%)

*SA *Sebaceous Adenoma*, SC *Sebaceous Carcinoma*, ka *Keratoacanthoma*, SCC *Squamous Cell Cancer*, BCC *Basal Cell Cancer

There was a male predominance (Male to Female (M:F) ratio: 1.6:1), which was most pronounced among patients with sebaceous (M:F = 1.7:1) compared to non-sebaceous skin lesions (M:F = 1.4:1). The median age for the onset of skin lesions was 55 years. The median age at diagnosis of sebaceous skin lesions was 55 years (28–84), and of non-sebaceous skin lesions 53 years (26–75). Notably, one patient with a strong family history of LS-associated cancers, had evidence of sebaceous hyperplasia from 17 years [14]. The median age at diagnosis of visceral malignancies was 47 (21–72). In a small but substantial minority (34.7%) of patients, the cutaneous lesion predated the visceral tumours by a median of 1 year (0–19). In those patients where LS was not immediately recognised, the interval between diagnosis of cutaneous and visceral tumours was prolonged, up to a median of 7 years (1–19).

### Immunohistochemistry and microsatellite testing

Two hundred and seventy-eight skin lesions from 171 patients who ultimately had molecularly confirmed LS were assessed by IHC and/or MSI testing. In lesions for which IHC (n = 253) was undertaken, 239 (94%) demonstrated MMR protein loss corresponding to their underlying germline genetic defects, but a minority of skin lesions (n = 14, 5%) were found to be MMR proficient, including 5 Sebaceous Adenomas, 2 keratoacanthoma, 2 SCC, 1 Melanoma and 4 others.

Of those 114 tumours for which MSI testing was undertaken, 103 tumours (90%) demonstrated MSI, but 11 skin lesions (10%) were MSS, including 6 SCC, 2 Sebaceous Adenomas, 1 Sebaceous Carcinoma, 1 keratoacanthomas, and 1 intradermal melanocytic nevus.

Of 91 skin lesions for which both MMR IHC and MSI testing were undertaken, discordance between the two tumour-based tests were noted in 10 tumours (11%) from 9 cases (13%) (Table 3).

**Table 3:** Skin lesions showing discordance between their IHC and MSI test results:

No	Case	Tumor	Genotype	IHC	MS
1	1	KA	*MSH2*	Intact	MSI-High
2	2	SA	*MSH6*	MSH2/MSH6 loss	Stable
3	2	SA	*MSH6*	MSH2/MSH6 loss	Stable
4	3	SC	*MSH2*	MSH2/MSH6 loss	Stable
5	4	SCC	*MSH2*	MSH2/MSH6 loss	Stable
6	5	SCC	*MLH1*	MLH1/PMS2 loss	Stable
7	6	SCC	*MSH2*	MSH2/MSH6 loss	Stable
8	7	SCC	*MSH2*	MSH2/MSH6 loss	Stable
9	8	SCC	*MSH2*	MSH2/MSH6 loss	Stable
10	9	Melanoma	*MLH1*	Intact	MSI-High

*IHC *Immunohistochemistry*, MSI *Microsatellite Instability

There were additional 50 sebaceous tumours from 49 molecularly confirmed LS patients in which the names of the genes and the details of the IHC testing were not determined in the papers. Forty-eight of these sebaceous tumours were reported to show MMRd (96%).

In the majority of sebaceous adenomas, sebaceous carcinomas, and sebaceomas, the patterns of MMR protein loss were consistent with their underlying germline defect: [111 (96%), 44 (100%), and 22 (100%), respectively]. Furthermore, most of them were MSI [32 (95%), 19 (95%), and 18 (100%), respectively]. On the other hand, 12 (86%) of the keratoacanthomas were reported to show consistent MMR protein loss, and 4 (80%) were MSI.

Seventy-four patients with molecularly confirmed LS developed SCC. Twenty-two SCCs were assessed for MMR deficiency by IHC, of which 20 (91%) were reported to have MMR protein loss consistent with their underlying genotype. Sixteen SCCs in patients with LS were tested for MSI, of which 10 (63%) were reported to demonstrate MSI. Tumour-based testing of BCCs in patients with LS was rarely undertaken, with 3 (9%) assessed by IHC; all of which were reported to demonstrate a consistent MMR protein loss (100%), and only one case (3%) was tested for MSI, and it was also found to be MSI (100%).

Another notable skin lesion reported in molecularly confirmed cases was melanoma (N = 14) with median age of diagnosis at 57 (Range: 26–68). IHC and MSI testing was undertaken on melanomas from only two patients with molecularly confirmed LS, both of which were found to be MSI-High, and one was MMRd. The details of IHC and MSI testing of the lesions from molecularly confirmed cases are shown in (Online Resource 4).

## Discussion

Prevention and/or early detection of cancer in patients with Lynch Syndrome is associated with increased survival [15]. The link between LS and sebaceous skin manifestations is well established [16]. The total number of MTS cases reported in the literature (n = 604) has increased by at least 200% in the last two decades compared to its total number in 1999, reflecting an increased awareness of MTS and LS, as well as an increase in the access to genetic testing [16].

In the general populations, non-sebaceous skin lesions, including SCC, BCC, melanomas, are widely studied and characterized. However, frequency and characteristics of sebaceous tumours, particularly benign sebaceous neoplasms, are poorly defined. On the contrary, in Lynch Syndrome patients, sebaceous tumours have been widely investigated for the last 50 years, whereas little attention has been given to non-sebaceous skin lesions. As a result, the exact incidence of different sebaceous tumours in the general population is not known, and according to available resources, they are considered rare. In a 9-year-retrospective study, Manonukul et al. found sebaceous tumours in only 2.34% of all skin lesion biopsies [17]. In a population-based study, Dores et al., using the (Surveillance, Epidemiology, and End Results) data, indicated an incidence rate of sebaceous carcinoma in the order of 0.11/100,000 person-years [18]. A more recent study by Sargen et al., using the same data, suggested an overall incidence of 2.4 cases per million [19, 20]. Non-sebaceous tumours, on the other side, including BCC, SCC, and to a lesser extent melanoma, are among the most common types of cancer in the general population, with estimated incidences of 158,934, 47,977, 15,332, respectively in 2019 in the UK [21].

Many authors postulate that the risk of squamous and other cutaneous malignancies in LS is under-recognised [10, 11]. Accordingly, in this systematic review, the most commonly reported lesions in the molecularly confirmed cases were sebaceous adenoma (43%), sebaceous carcinoma (27%), sebaceoma (13%), and keratoacanthoma (16%). However, in addition to those skin lesions, which have already been linked to LS, there were high frequencies of SCC (23%) and BCC (10%).

The male to female ratio in the cases, similar to skin cancers in the general population, was higher in males compared to females. However, the age of onset of the skin tumours was much lower compared to their sporadic counterparts. The median age of onset of sebaceous carcinomas, SCC, and BCC in the patients were 56, 55, 53 years, compared to 73, 75, 68 years, respectively, in the general population [22, 23]. This gap in their ages of onset suggests a potential role for Lynch Syndrome in the pathogenesis of these skin tumours.

Non-sebaceous cutaneous malignancies are common, with risk increased by sun exposure, particularly among patients with Fitzpatrick Type I/II skin types. A number of hereditary disorders associated with melanoma and non-melanoma skin cancers have been identified. Many of the heritable conditions associated with an increased risk of squamous and/or basal cell skin cancers are rare, recessive or X-linked disorders associated with DNA repair defects—such disorders include Xeroderma Pigmentosum, Fanconi Anaemia, Bloom Syndrome, Rothmund Thomson Syndrome, Werner Syndrome or Dyskeratosis Congenita [24]. Other rare causes of basal cell skin cancer include autosomal dominant Gorlin Syndrome, Bazex-Dupré-Christol Syndrome or Rombo Syndrome [24]. These syndromic disorders are typically associated with other non-malignant features; and some, particularly those recessive disorders associated with DNA repair defects, will be evident from early childhood [24]. Lynch Syndrome, in contrast, has few non-malignant manifestations, and is easily missed—with a reported 95% of affected individuals unaware of their genetic diagnosis [1, 6]. Diagnosis of LS therefore relies on clinicians being alert to family history and tumour features consistent with this autosomal dominant disorder.

In this cohort, although SCC and BCC were noted in patients with molecularly confirmed LS, few of the tumours were assessed by IHC or MSI, and attribution of skin cancer risk to the underlying genotype cannot be confirmed. Furthermore, compared to sebaceous skin lesions, higher numbers of SCC (64%) and BCC (86%) were occurring in sun exposed areas, suggesting an environmental risk factor. Melanomas have been linked to LS to a much lesser extent compared to SCC and BCC in the literature. However, 8 of the molecularly confirmed LS cases in this review had histories of melanoma. Family history of melanoma was also reported in 6 other molecularly confirmed cases. In addition to cutaneous melanomas, there are reports of ocular melanomas in LS patients in the literature [25, 26].

Of those cutaneous tumours that were assessed by MMR IHC and/or MSI in molecularly confirmed cases, the majority were MMRd or MSI-high, or both. However, the sensitivity of MMR IHC was higher compared to MSI testing, particularly among keratoacanthoma and SCC. This does not appear to be limited to LS—in a small case series of unselected apparently sporadic cutaneous SCC, microsatellite instability was rare (1 of 22), but MMR deficiency as defined by loss of one or more proteins detected by IHC, more common (4 of 22) [27]. It is well-recognised that the relative sensitivity of microsatellite testing in tumours of patients with LS is variable across tissue types, and greatly influenced by neoplastic cell content in the sample, such that IHC may be preferable in assessment of extracolonic malignancies, or in tumours of low cellularity [10, 28]. Recently, Ykema et al. reported ten SCCs in seven molecularly confirmed Lynch Syndrome cases, all of which were IHC deficient (100%), but only three of 9 assessed for microsatellite instability (33%) demonstrated MSI [10]. Sowter et al., in his response to Ykema et al. study, strengthened this link by testing nine SCCs from seven other molecularly confirmed cases (data outside of the timeframe of this review), and found that eight tumours were MSI-H (89%) [12]. Although in both studies the total number of SCCs and patients were small, the findings of immunohistochemically demonstrated MMR deficiency and/or MSI suggests that the underlying diagnosis of Lynch Syndrome is clinically relevant to the pathogenesis of such cancers. The discordance between MMR immunohistochemistry and MSI testing in LS-associated non-colorectal samples reflects discordance observed by other authors.

Basal and squamous cell cancers are much more common in the general population than sebaceous neoplasms/keratoacanthoma, limiting the feasibility of universal screening by MMR IHC/MSI for non-sebaceous malignancy. Universal screening of sebaceous malignancies may be more readily achievable, but the likelihood of MMRd or MSI being attributable to underlying LS in isolated cases without personal/family history of other LS cases is very low [29]. Rather than universal screening, judicious application of MMR IHC and/or MSI testing in patients with any type of skin cancers and suspicious family histories and/or earlier than expected age at diagnosis and absence of other relevant risk factors, may facilitate detection of LS, and in some, may predate and help manage risk of visceral malignancy.

Immune checkpoint inhibitors have been shown to have activity in MSI-H/ MMRd cancers regardless of site of origin [30]. In 2017, the Food and Drug Administration (FDA) approved pembrolizumab for advanced MSI-H/MMRd malignancies, this was the first tissue-agnostic drug approval for cancer by the FDA [31].

More recently, the anti PD-1 agent cemiplimab has shown efficacy in both advanced cutaneous SCC and BCC [32, 33]. This has led to drug approval by the European Medicines Agency and FDA for both indications, meaning patients can access immunotherapy irrespective of MMR/MSI status [34–36].

## Limitations

Few of the papers in this review provided comprehensive detail of the tumour and/or germline testing undertaken. In order to capture as wide a breadth of published literature as possible, we included all published literature, such that there is variability in the type of study and nature of data collection in the studies included in this review, precluding meaningful meta-analysis of data. The significant majority of published literature relates to single case reports. Furthermore, determining the frequency of skin lesions and their characteristics were limited to the molecularly confirmed Lynch Syndrome cases. As a result, there could be other skin lesions that are associated with Lynch Syndrome and have not been described in this paper.

## Conclusion

Skin lesions reported in patients with Lynch syndrome include SCC and BCC as well as sebaceous adenoma, sebaceous carcinoma, sebaceoma and keratoacanthoma, but further large-scale prospective studies, with robust paired tumour and germline assessments, are required to confirm causal association between non-sebaceous malignancy and Lynch Syndrome. In the meantime, patients should be provided with general advice to manage the risk of cutaneous malignancies—taking care to avoid sunburn or exposure to excess UV irradiation (sunbeds), using high factor sun cream, and seeking prompt advice when they develop any new skin lesions. They should also be provided with advice regarding vitamin D replacement as necessary. Dermatologists should be alert to the relevance of a family history of colorectal, endometrial or other Lynch Syndrome associated cancers in individuals presenting with non-sebaceous as well as sebaceous neoplasms, with assessment of MMR by immunohistochemistry and/or MSI testing, and onward germline testing or referral to Clinical Genetics undertaken as required.

## Supplementary Information

Below is the link to the electronic supplementary material.

Supplementary file1 (XLSX 46 kb)

Supplementary file2 (XLSX 20 kb)

Supplementary file3 (XLSX 15 kb)

Supplementary file4 (XLSX 13 kb)

## Data Availability

The datasets generated during and/or analysed during the current study are available from the corresponding author on reasonable request.
